# Hybrid antiferroelectric–ferroelectric domain walls in noncollinear antipolar oxides

**DOI:** 10.1038/s41565-026-02139-8

**Published:** 2026-04-15

**Authors:** Ivan N. Ushakov, Mats Topstad, Muhammad Z. Khalid, Niyorjyoti Sharma, Christoph P. Grams, Ursula Ludacka, Jiali He, Kasper A. Hunnestad, Mohsen Sadeqi-Moqadam, Julia Glaum, Sverre M. Selbach, Joachim Hemberger, Petra Becker, Ladislav Bohatý, Amit Kumar, Jorge Íñiguez-González, Antonius T. J. van Helvoort, Dennis Meier

**Affiliations:** 1 Department of Materials Science and Engineering, Norwegian University of Science and Technology (NTNU), Trondheim, Norway; 2 Department of Physics, Norwegian University of Science and Technology (NTNU), Trondheim, Norway; 3 Centre for Quantum Materials and Technologies (CQMT), Queen’s University Belfast, Belfast, UK; 4 Institute of Physics II, University of Cologne, Cologne, Germany; 5 Department of Electronic Systems, Norwegian University of Science and Technology (NTNU), Trondheim, Norway; 6 Institute of Geology and Mineralogy, University of Cologne, Cologne, Germany; 7 Smart Materials Unit, Luxembourg Institute of Science and Technology (LIST), Esch/Alzette, Luxembourg; 8 Department of Physics and Materials Science, University of Luxembourg, Belvaux, Luxembourg; 9 Faculty of Physics and Center for Nanointegration Duisburg-Essen (CENIDE), University of Duisburg-Essen, Duisburg, Germany; 10 Research Center Future Energy Materials and Systems, Research Alliance Ruhr, Bochum, Germany

**Keywords:** Ferroelectrics and multiferroics, Electronic properties and materials

## Abstract

Antiferroelectrics are emerging as advanced functional materials with unique electric properties enabled by the antipolar arrangement of their electric dipoles. Additional functionalities and novel physical nanoscale phenomena are expected in systems with noncollinear antipolar dipole structures. Here we demonstrate how the onset of antiferroelectricity in K_3_[Nb_3_O_6_∣(BO_3_)_2_] drives noncollinear ordering of electric dipole moments, which leads to unusual hybridization of antiferroelectric and ferroelectric responses. Besides the double-hysteresis loop common to antiferroelectrics, a pronounced piezoresponse and electrically switchable hybrid domains are observed using scanning probe microscopy. Scanning transmission electron microscopy shows that the domains are separated by atomically sharp and micrometre-long charged domain walls with inseparably entangled discontinuities in the antiferroelectric and ferroelectric orders. Hybrid antiferroelectric–ferroelectric responses are expected in a wide range of noncollinear systems.

## Abstract (web summary)

Hybrid antiferroelectric–ferroelectric domains and domain walls in K_3_[Nb_3_O_6_(BO_3_)_2_] are reported, enabled by noncollinear ordering of electric dipole moment.

## Main

**Fig. 1:**
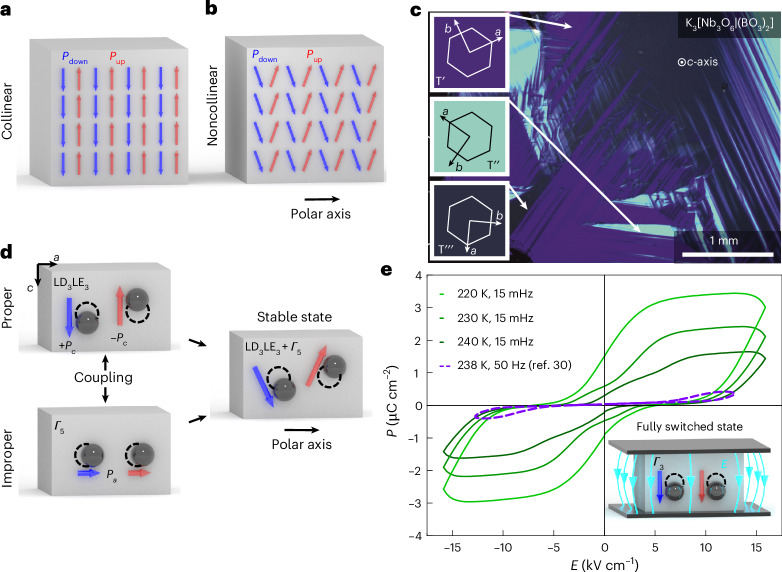
Noncollinear antipolar order in K_3_[Nb_3_O_6_∣(BO_3_)_2_]. **a**, In conventional antiferroelectrics, the electric dipole moments (blue and red arrows) are antiparallel, which gives zero net polarization and conserves inversion symmetry. **b**, A canting of the antipolar order reduces the symmetry and leads to the formation of a polar axis, enabling additional physical properties that are symmetry forbidden in collinear systems. **c**, Polarized light microscopy of the K_3_[Nb_3_O_6_∣(BO_3_)_2_] (001) sample face reveals three ferroelastic 120° twin domain states, labelled as ${\mathrm{T}}^{{\prime} },{\mathrm{T}}^{{\prime \prime} }$ and ${\mathrm{T}}^{{\prime\prime} {\prime} }$. The respective crystallographic orientations are sketched as insets with the *a*-axis being the polar axis. **d**, In K_3_[Nb_3_O_6_∣(BO_3_)_2_], noncollinear antipolar order arises from the coupling of antipolar (LD_3_LE_3_) and polar (*Γ*_5_) modes, defining the system as a proper antiferroelectric, improper ferroelectric (and ferroelastic) material. **e**, For electric fields, *E* (15 mHz), applied along the *c*-axis (perpendicular to the polar axis), a double-hysteresis loop opens up for *P*(*E*) as the temperature decreases, consistent with previous experiments conducted at higher frequencies (purple dashed line, adapted from ref. ^30^). The switching behaviour is due to a polar instability (*Γ*_3_) parallel to the antipolar direction as sketched in the inset, leading to an electric-field-induced polar state.

In 1928, Louis Néel established the fundamentals of antiferromagnetism^1^. About 20 years later, Charles Kittel expanded the concept towards antiferroelectrics, defining them as systems with lines of spontaneously polarized ions pointing in antiparallel directions^2^ (Fig. 1a). Owing to the absence of a macroscopic polarization, antiferroelectrics were initially considered to be of limited technological interest. This perception has changed, and today, they represent promising functional materials for, for example, solid-state refrigeration and energy applications^3–5^. Additional functionality arises from their local structure, ranging from unusual domain walls to topological vortex textures^6–8^.

In principle, antiferroelectricity is compatible with polar point groups, allowing for noncollinear order as sketched in Fig. 1b (refs. ^9–11^). Examples of systems with noncollinear antipolar order include Gd_2_(MoO_4_)_3_ (ref. ^12^), BiCu_*x*_Mn_7−*x*_O_12_ (ref. ^13^), La-doped Pb(Zr,Sn,Ti)O_3_ (ref. ^14^) and organic salts^15^. Analogous to noncollinear spin textures, a canting of electric dipoles can reduce the symmetry and has been predicted to give rise to Dzyaloshinskii–Moriya-like interactions^16^ and unlock exotic otherwise symmetry-forbidden properties^17^. In contrast to magnetic materials, however, the concept of noncollinearity in antiferroelectrics is much less explored and canting-related effects, in particular on the local domain level, remain to be demonstrated.

Here we study domains and domain walls in noncollinear antiferroelectric potassium niobate borate K_3_[Nb_3_O_6_∣(BO_3_)_2_] using piezoresponse force microscopy (PFM) and tomographic atomic force microscopy. Three-dimensional (3D) mapping reveals micrometre-long planar charged and neutral walls, and our symmetry and density functional theory (DFT) analysis show that an antiferroelectric instability drives the noncollinear electric dipole order. Ferroelectricity and ferroelasticity arise as secondary (improper) effects, which are inseparably entangled with the (primary) antiferroelectric order. As a consequence, atomically sharp hybrid domain walls form with non-trivial electric-field responses, which we image by scanning transmission electron microscopy (STEM) and electrostatic force microscopy (EFM), respectively.

## Origin of noncollinear antiferroelectricity

K_3_[Nb_3_O_6_∣(BO_3_)_2_] crystals are grown as described in ref. ^18^. The crystals are hexagonal at growth temperature (space group *P*$\bar{6}2m$, 189)^19^ and orthorhombic at room temperature (space group *P*2_1_*ma*, 26)^18^, with a quadruplicated unit cell volume (Supplementary Fig. 1). The phase transition (Aizu species $\bar{6}2mFm2m$) leads to three polar 120° ferroelastic domain states (${\mathrm{T}}^{{\prime} }, {\mathrm{T}}^{{\prime \prime} }$ and ${\mathrm{T}}^{{\prime\prime} {\prime} }$)^20–22^. At the micrometre scale, the ferroelastic domains can readily be visualized optically^23^ (Fig. 1c), whereas their nanoscale physics remain to be explored.

**Extended Data Fig. 1:**
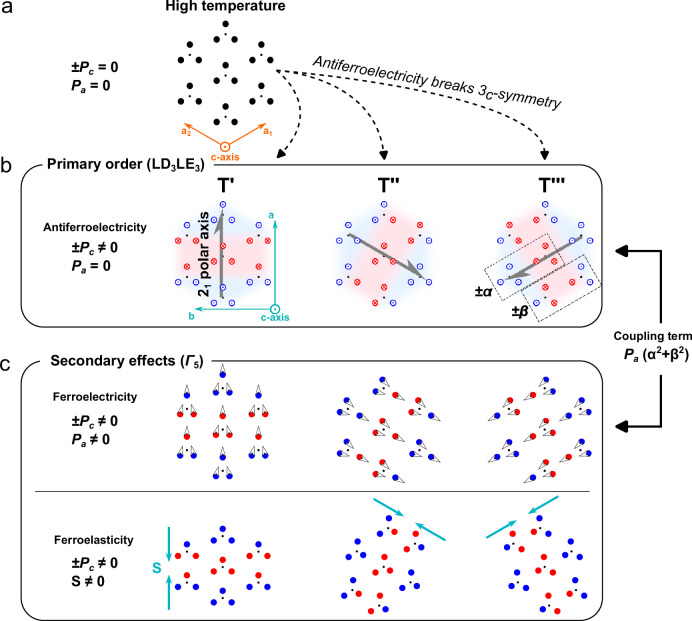
Order parameters and their coupling in K_3_[Nb_3_O_6_∣(BO_3_)_2_]. **a**, Positions of the Nb atoms in the parental high-temperature phase. The structure has a threefold rotational symmetry around the *c*-axis. **b**, When the antiferroelectric order parameter is activated, the threefold symmetry breaks, leading to the formation of three polar orientational domains ${\mathrm{T}}^{{\prime} }, {\mathrm{T}}^{{\prime \prime} }$ and ${\mathrm{T}}^{{\prime\prime} {\prime} }$. These domains are purely antiferroelectric as the LD_3_LE_3_ mode does not induce ferroelectricity or ferroelasticity. **c**, Activating ferroelectricity (*Γ*_5_), and/or ferroelasticity, does not break additional symmetries. The latter are coupled to antiferroelectricity through a linear-quadratic term and cannot exist independently. We note that the B atoms (not shown) dominate the ferroelectric displacement, whereas Nb atoms dominate the antipolar displacement.

DFT calculations (Supplementary Note 1, Supplementary Table 1 and Supplementary Fig. 2) reveal an antipolar instability (LD_3_LE_3_ mode) of the high-temperature phase, with local electric dipoles forming along the *c*-axis (Fig. 1d). By performing a customary symmetry analysis^24,25^, we conclude that the LD_3_LE_3_ mode is the primary order parameter driving the phase transition to the room-temperature phase. Because of the threefold symmetry in the hexagonal high-temperature phase, however, a pure antipolar arrangement cannot be geometrically satisfied (Extended Data Fig. 1 and Supplementary Fig. 1). The LD_3_LE_3_ mode breaks the threefold symmetry and introduces a polar axis, which leads to three antiferroelectric domain states. As a consequence of the symmetry breaking, additional secondary modes are enabled that do not further reduce the symmetry. In fact, we find a secondary mode of polar character and symmetry *Γ*_5_ that acts as an improper order and leads to ferroelectricity and ferroelasticity (Fig. 1d, Extended Data Fig. 1 and Supplementary Table 1). Thus, the room-temperature phase—where both the LD_3_LE_3_ and *Γ*_5_ modes coexist—can be characterized as proper antiferroelectric and improper ferroelectric, with noncollinear (canted) antipolar order. The coexisting orders are inseparably entangled, analogous to spin-driven improper ferroelectrics, where the coupling between (proper) antiferromagnetic and (improper) ferroelectric properties leads to intriguing correlation effects, such as hybrid domains, which exhibit both magnetic and electric characteristics^26^. Our calculations give polarization components *P*_*a*_ ≈ 0.03 μC cm^−^^2^ and ±*P*_*c*_ ≈ 19 μC cm^−^^2^, where *P*_*a*_ is the net spontaneous polarization, and ±*P*_*c*_ are the alternating components of the electric dipoles per half unit cell volume (Fig. 1d and Supplementary Fig. 1). For reference, the resulting spontaneous polarization, *P*_*a*_, is about 500 times smaller than in BaTiO_3_ (≈ 15 μC cm^−^^2^)^27^ and 1–2 orders of magnitude smaller than in other improper ferroelectrics, such as Cu_3_B_7_O_13_Cl (≈ 1.8 μC cm^−^^2^)^28^ and Gd_2_(MoO_4_)_3_ (≈ 0.2 μC cm^−^^2^)^29^.

Importantly, because of the antipolar components ±*P*_*c*_, K_3_[Nb_3_O_6_∣(BO_3_)_2_] shows a double-hysteresis loop for electric fields along the *c*-axis^30^, that is, perpendicular to the polar *a*-axis. Such double-hysteresis loops are commonly considered a hallmark of antiferroelectricity^4,31,32^. Figure 1e shows respective low-frequency (15 mHz) *P*_*c*_(*E*) measurements (Methods) together with literature data (50 Hz)^30^. Within the electric-field range of our experiment, we observe non-saturated double-hysteresis loops with a maximum electric-field-induced polarization along the antipolar *c*-axis of about 3.5 μC cm^−^^2^ (*T* = 220 K).

On the basis of our DFT and symmetry analysis, we attribute the switching behaviour to an interplay between the primary antipolar (LD_3_LE_3_) mode (*E* = 0) and a field-aligned state (*Γ*_3_), approaching a field-aligned state parallel (+*E*) or antiparallel (−*E*) to the antipolar *c*-axis (inset in Fig. 1e), reverting to noncollinear antipolar order as the electric field is removed (*Γ*_5_ is not involved in the double-hysteretic switching).

## Hybrid antiferroelectric–ferroelectric domain walls

**Fig. 2:**
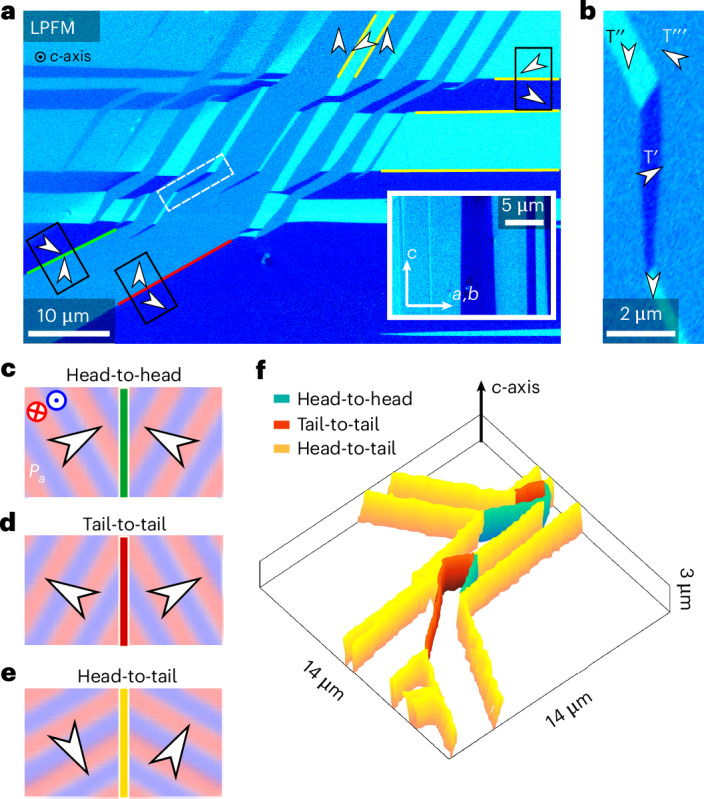
Coexistence of neutral and charged domain walls. **a**, Lateral PFM on a (001) sample face, plotted as $A\cos \phi$, where *A* is the amplitude and $\phi$ is the phase. Inset: lateral PFM of a surface with the *c*-axis in-plane. **b**, Close-up of the region marked by the dashed white rectangle in (**a**). Arrows indicate the strongest piezoresponse directions in the three types of domain (${\mathrm{T}}^{{\prime} }, {\mathrm{T}}^{{\prime \prime} }$ and ${\mathrm{T}}^{{\prime\prime} {\prime} }$) as labelled in **b**. This direction coincides with the direction of *P*_*a*_. **c**–**e**, Three domain wall types are observed: positively charged head-to-head (green) (**c**), negatively charged tail-to-tail (red) (**d**) and neutral head-to-tail (yellow) (**e**) walls. The coexisting antipolar order (±*P*_*c*_) is indicated by pink (−*P*_*c*_) and violet (+*P*_*c*_) colours. **f**, Tomographic atomic force microscopy data showing the 3D structure of the different types of domain wall. Source data

Figure 2a shows a PFM image (lateral signal, LPFM) from a surface perpendicular to the *c*-axis, revealing a pronounced piezoresponse with three contrast levels. This behaviour is fundamentally different from classical collinear antiferroelectrics, such as prototypical PbZrO_3_, where piezoelectricity in the antiferroelectric state is forbidden by symmetry^33,34^.

By performing vector PFM (Supplementary Note 2), we determine the orientation of the polar *a*-axis within the different domains. This orientation is identical to the direction in which the piezoresponse has its maximum (Supplementary Note 1) and gives the direction of *P*_*a*_ as indicated by white arrows in Fig. 2a. We identify three domain states with *P*_*a*_ changing by 120° across the domain walls, consistent with the expected ferroelastic twin domain states ${\mathrm{T}}^{{\prime} }, {\mathrm{T}}^{{\prime} }$ and ${\mathrm{T}}^{{\prime\prime} {\prime} }$, and the stripe-like domain structure observed at larger length scales (Fig. 1c). The nanoscale domain structure, however, is more complex than suggested by the optical experiments, exhibiting chevron-like patterns with sequences of alternating domain states, some ending in narrow needle-shaped domains (Fig. 2b). This behaviour is classical to ferroelastics, allowing the material to minimize mechanical strain^35,36^.

The domain walls show different polarization configurations with respect to *P*_*a*_, including head-to-head and tail-to-tail (minority) and head-to-tail (majority) arrangements (Fig. 2c–e). PFM images gained on a perpendicular surface (inset in Fig. 2a) and PFM tomography (Fig. 2f and Supplementary Note 2) reveal that all walls propagate parallel to the *c*-axis, forming planar two-dimensional (2D) systems. This is consistent with ferroelastic symmetry considerations^37,38^; however, the extension of head-to-head and tail-to-tail walls over several micrometres is unusual, because they represent polar discontinuities that carry bound surface charges $\pm 2{P}_{a}\cos 3{0}^{\circ }=\pm 0.05$ μC cm^−^^2^, which makes them energetically costly. So far, charged planar walls with comparable physical dimensions have only been observed in boracites^39^. In the case of the boracites, however, their injection requires the application of point pressure, whereas the charged head-to-head and tail-to-tail walls naturally form at *T*_*c*_ in K_3_[Nb_3_O_6_∣(BO_3_)_2_]^40^ (Supplementary Fig. 6). The domain walls in Fig. 2 are thus fundamentally different from previously studied interfaces in ferroelectrics, antiferroelectrics and mixed systems with coexisting orders at the bulk level^14,41^, as *P*_*a*_ and ±*P*_*c*_ are inseparably entangled, leading to a discontinuity in both the antiferroelectric and ferroelectric orders across the walls (Fig. 2c–e). Thus, analogous to domain walls in spin-driven ferroelectrics^26^, a description of the walls in terms of ‘antiferroelectric walls’ or ‘ferroelectric walls’, or even ‘walls with coexisting antiferroelectric and ferroelectric orders’, is not adequate, classifying them as hybrid antiferroelectric–ferroelectric (and ferroelastic) domain walls. It is the primary symmetry-breaking order parameter (proper antiferroelectric) that drives the formation of these walls, whereas the secondary order parameters are energetically less relevant, co-determining only specific features, such as shape and orientation^37^ or the number of charged versus neutral walls^37,42^.

**Fig. 3:**
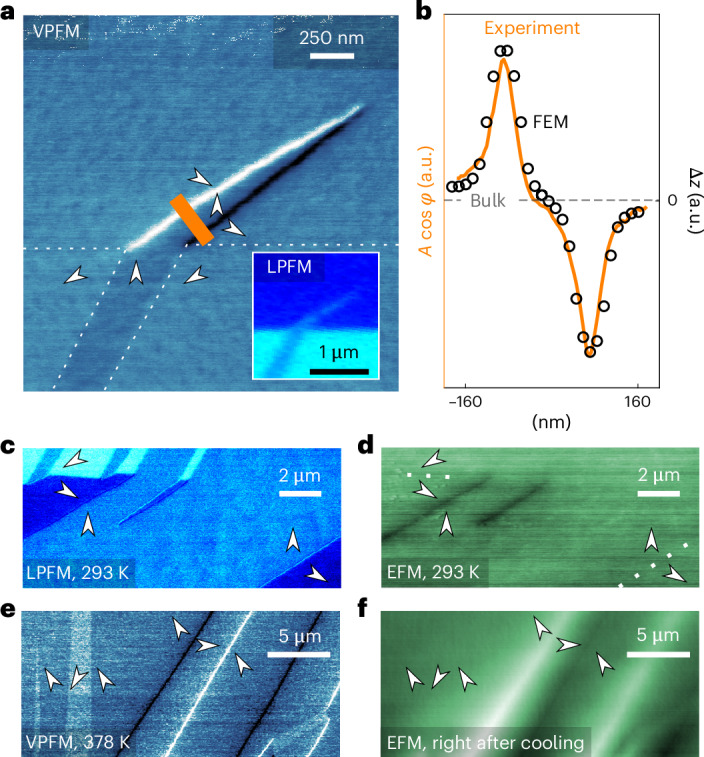
Electromechanical and electrical domain wall properties. **a**, Vertical resonance PFM on a (001) sample face (plotted as $A\cos \phi$) shows a pronounced piezoelectric response at the head-to-head and tail-to-tail domain walls. Corresponding LPFM data are presented in the inset of **a**. **b**, Profile of the head-to-head and tail-to-tail domain walls, as indicated by the orange line in **a** and corresponding FEM simulation of the static displacement (Δ*z*) in the *c*-direction. The dashed line represents the average signal in the bulk. **c**,**d**, The comparison of LPFM ($A\cos \phi$) (**c**) and EFM (amplitude, (001) face) (**d**) scans at room temperature shows that the strongest electrostatic response arises at the head-to-head domain walls. **e**,**f**, Complementary VPFM- (**e**) and EFM-based (**f**) annealing experiments at elevated temperature, however, reveal equally pronounced electrostatic forces at head-to-head and tail-to-tail domain walls, indicating that the asymmetric EFM response at room temperature is due to extrinsic screening effects. Source data

Next, we analyse the local electronic properties at these hybrid domain walls. At the local scale, K_3_[Nb_3_O_6_∣(BO_3_)_2_] is highly resistive with no detectable differences between domains and domain walls (Supplementary Fig. 7) consistent with its large band gap (colourless crystals). We thus focus on the local electromechanical and electrostatic properties in Fig. 3. Figure 3a shows a high-resolution PFM image (vertical signal, VPFM) of one of the needle-shaped domains shown in the inset. A pronounced electromechanical response is measured at the head-to-head (bright) and tail-to-tail (dark) walls, which distinguishes them from the twin domains and the neutral head-to-tail walls.

To understand the anomalous local response, we perform finite element modelling (FEM; Supplementary Note 1). Consistent with the piezoresponse at charged ferroelectric walls^43^, the model shows that the shear strain changes sign at the hybrid walls, leading to an upward displacement (bright) at the head-to-head walls and a downward displacement (dark) at the tail-to-tail walls. In Fig. 3b, we present the simulated contraction/expansion for neighbouring head-to-head and tail-to-tail domain walls. The simulation is in excellent agreement with our cross-sectional VPFM data (Fig. 3a) and the direction of the polar axis determined by vector PFM. We thus conclude that the electromechanical response at the hybrid walls originates from counteracting shear strains, enabled by the primary LD_3_LE_3_ mode.

The electrostatic properties associated with the hybrid antiferroelectric–ferroelectric walls are presented in Fig. 3c,d. By correlating LPFM and EFM (Methods) data, we find that the positively charged head-to-head walls exhibit an electrostatic response different from the domains, being visible as straight dark lines in Fig. 3d. By contrast, no signature is resolved in this EFM scan at the negatively charged tail-to-tail and the neutral head-to-tail walls (although occasionally resolved in other scans with four to five times smaller magnitude than for the head-to-head walls; Supplementary Fig. 8). We note that the surface potential variations do not come from topographical contributions (Supplementary Fig. 9). The observation that the surface potential varies mainly at the position of the positively charged head-to-head walls suggests that the negative bound charges at the tail-to-tail walls are screened more efficiently^44–49^. To clarify the screening mechanism, we conduct experiments at elevated temperature (Fig. 3e,f and Supplementary Fig. 10). Right after heating or cooling across the dielectric anomaly that occurs at about 383–393 K (ref. ^50^), both head-to-head and tail-to-tail walls exhibit equally pronounced EFM contrasts of opposite sign, consistent with their bound charge. After about 30 minutes, however, only head-to-head walls retain a contrast, analogous to Fig. 3d. This effect, together with the observation that the domain wall contrasts invert in heating and cooling experiments (Supplementary Fig. 10), leads us to the conclusion that the asymmetry in electrostatic potential at head-to-head and tail-to-tail walls is due to extrinsic adsorbates that screen the positive and negative bound charges with different efficiencies^48,49^.

## Atomic-scale domain wall structure

**Fig. 4:**
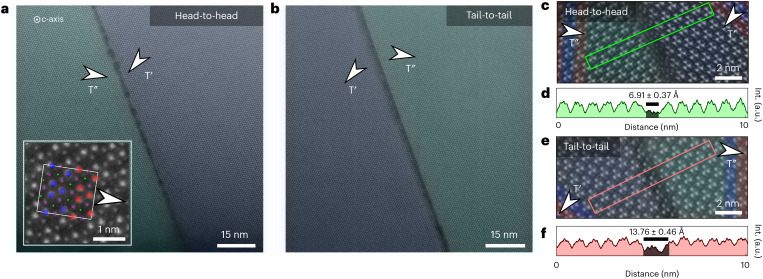
Atomic structure of hybrid antiferroelectric–ferroelectric domain walls. **a**, False-coloured HAADF-STEM image of the (001) sample face showing a head-to-head domain wall. Inset: a high-resolution image of the Nb atoms (highlighted red and blue) gained on a different, FIB-prepared sample. **b**, Same as in **a** for a tail-to-tail domain wall. **c**, High-resolution HAADF-STEM image of a head-to-head domain wall. False colours (red and blue) illustrate how the antipolar order (obtained via correlated PLM and PFM data; Extended Data Fig. 1 and Supplementary Fig. 11) changes across the domain wall. **d**, Profile from **c** showing the local drop in intensity (int.), which gives an estimate for the width of the head-to-head domain wall as discussed in the main text. **e**,**f**, Same as in **c** and **d**, respectively, for a tail-to-tail domain wall. Source data

High-resolution images of the atomic-scale structure at head-to-head and tail-to-tail walls are presented in Fig. 4. The high-angle annular dark-field STEM (HAADF-STEM) images are recorded viewing along the *c*-axis with the bright dots representing the Nb atomic columns (inset in Fig. 4a). The domain walls are visible as straight dark lines in Fig. 4a,b, whereas a regular lattice structure is observed away from the walls. Across the walls, the polar axis rotates by 120°, forming nominally charged head-to-head (Fig. 4a) and tail-to-tail (Fig. 4b) configurations, as confirmed by correlated PLM–PFM–TEM measurements (Supplementary Fig. 11). Importantly, the direction in which the antipolar displacement of Nb atoms is modulated changes together with the polar axis from one domain to the next as indicated by white arrows. The change in the antipolar order is highlighted in Fig. 4c,e, where blue and red colours indicate polarization up (+*P*_*c*_) and down (−*P*_*c*_), respectively. The orientational changes in the antiferroelectric and ferroelectric orders go hand in hand with a structural phase shift (locally ≈ 210°; see Supplementary Fig. 12 for more information). In contrast to reported combinations of out-of-phase boundaries and charged domain walls^51,52^, there is no evidence for pronounced compositional variations at the hybrid walls in K_3_[Nb_3_O_6_∣(BO_3_)_2_]. Instead, we attribute the domain-wall-related intensity drop in the HAADF-STEM data to a structural displacement by a fraction of the unit cell along the *c*-axis (Supplementary Fig. 13). This conclusion is supported by the absence of pinning effects and the formation of new domain wall patterns after heating above *T*_*c*_. As shown in Fig. 4d,f and Supplementary Fig. 14, the intensity drop width (*d*_*w*_) varies locally between 6.91 ± 0.37 Å and 18.50 ± 0.94 Å for the head-to-head wall, whereas the tail-to-tail wall is rather smooth with *d*_*w*_ = 13.76 ± 0.46 Å. The data show that the lower wall width (≈ 7 Å) is similar to the size of the Nb trimers in K_3_[Nb_3_O_6_∣(BO_3_)_2_] and comparable to charged domain walls in other improper ferroelectrics such as ErMnO_3_ (ref. ^53^). We note that we do not resolve phase shifts or intensity drops associated with head-to-tail walls, which implies that structural defects need to form at their connection points with head-to-head and tail-to-tail walls to accommodate the phase difference.

The HAADF-STEM data show the hybrid nature of the domain walls at the atomic scale, exhibiting characteristic discontinuities in both the antiferroelectric and ferroelectric displacement patterns. Independent of their charge state, the hybrid antiferroelectric–ferroelectric walls form atomically sharp interfaces with a lateral extension of several micrometres (Fig. 2) and a width in the sub-unit-cell regime (Fig. 4).

## Electric-field control of hybrid domain walls

**Fig. 5:**
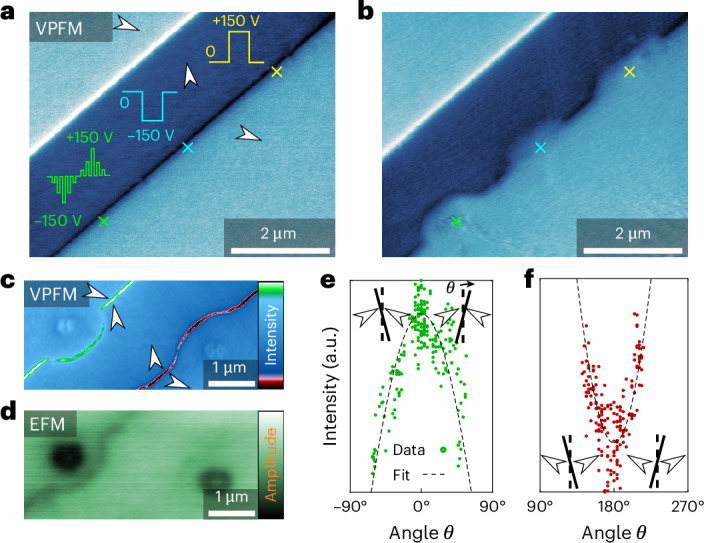
Local switching behaviour at head-to-head and tail-to-tail domain walls. **a**, Localized electric d.c. and a.c. fields of up to ±150 V are generated by the probe tip and applied close to the tail-to-tail wall at the positions marked by green (a.c. ±150 V), cyan (d.c. −150 V) and yellow (d.c. +150 V) crosses (VPFM, $A \cos \phi$). **b**, Independent of the polarity of the applied voltage, the tail-to-tail wall moves in the direction of the neighbouring head-to-head wall (VPFM, $A \cos \phi$). **c**, Vice versa, head-to-head walls move in the direction of the nearest tail-to-tail wall when an electric field is applied (VPFM, $A \cos \phi$ averaged over several scans). **d**, EFM (amplitude) data collected at the same position as in **c** after application of the electric field demonstrates that the domain-wall-related electrostatic anomaly (dark contrast) moves together with the domain wall. **e**, The analysis of the VPFM signal in the curved section of the head-to-head wall in **c** reveals that the piezoresponse varies along with the domain wall orientation, showing as sinusoidal relationship between the magnitude of piezoresponse and the orientation angle *θ*. The dashed line represents a sinusoidal fit. **f**, Same as in **e** for the tail-to-tail wall. Source data

To explore the general possibility to control the position of the hybrid antiferroelectric–ferroelectric domain walls, we investigate their response to local electric field poling (Fig. 5). Figure 5a,b shows that the position of individual domain walls can be controlled by electric fields, exemplified for the case of a tail-to-tail wall. PFM images are recorded before (Fig. 5a) and after (Fig. 5b) applying d.c. or a.c. bias voltages (Supplementary Fig. 15) at the positions marked by the crosses. We observe that, independent of the sign of the applied bias voltage, the tail-to-tail wall moves towards its nearest head-to-head counterpart. The same also applies for the head-to-head walls as shown in Fig. 5c and when the tip is placed between the domain walls (Supplementary Fig. 16).

One possible driving mechanism for this unusual switching behaviour is the electric field acting on the secondary strain-capable polar *Γ*_5_ mode, which interacts with the domain walls owing to their ferroelastic nature. We consistently find that neighbouring head-to-head and tail-to-tail walls approach each other and can even annihilate in poling experiments (Supplementary Fig. 17), indicating that associated phase shifts compensate each other, consistent with their stress-promoted annihilation observed in optical experiments on larger length scales^40^.

Importantly for this study, Fig. 5a,b shows that tail-to-tail and head-to-head walls are spatially mobile, readily moving by several hundreds of nanometres under the applied electric field of ≈6×10^4^ kV cm^−1^. For reference, this value is about one order of magnitude larger than the electric fields required for driving local displacements of (neutral) domain walls in improper ferroelectric ErMnO_3_ (ref. ^54^). We note that the charged walls are unaffected by electron-beam irradiation in HAADF-STEM (Supplementary Fig. 18), suggesting a lower mobility than conventional domain walls in classical antiferroelectrics such as PbZrO_3_, which were observed to move during lattice imaging^6,7^. EFM data gained at the poled position (Fig. 5d) reveals that the electrostatic signature moves along with the walls, corroborating that it is an intrinsic feature. Similar to the EFM scan in Fig. 3d, the head-to-head wall exhibits a pronounced variation in surface potential, whereas only a very weak EFM signal is measured at the new position of the tail-to-tail wall (dark spots in the EFM map mark the positions where the biased tip was placed). Head-to-tail walls are in general immobile when no head-to-head and tail-to-tail domain walls are nearby (Supplementary Fig. 19). However, increased mobility of head-to-tail walls is observed in the vicinity of head-to-head or tail-to-tail walls and the meeting points with them. This observation is consistent with the aforementioned phase mismatch between charged and neutral walls and the formation of structural defects that destabilize the head-to-tail walls.

In contrast to the straight domain walls in the unpoled state (Fig. 3a), the evaluation of the VPFM data (Supplementary Note 2) in Fig. 5c reveals that the electromechanical response is no longer constant along the wall after poling. As shown in Fig. 5e,f, in the curved wall segment, the piezoresponse continuously varies together with the charge state of the wall ($\propto 2{P}_{a}\cos \theta$), making it highly tunable. A correlation between charge state and piezoresponse was previously reported for domain walls in improper ferroelectric HoMnO_3_ (ref. ^55^), but it was studied only for as-grown domain walls without showing the possibility to control the local piezoresponse by changing the wall orientation.

Our analysis of the local electromechanical properties demonstrates that the piezoresponse of individual domain walls can be tuned, going from discrete softness states^56^ to a continuous set of states with gradually varying softness with the domain wall orientation as control parameters.

## Conclusion

We observed hybrid antiferroelectric–ferroelectric domain walls with unusual structures and responses, and showed that their formation is driven by inseparably entangled discontinuities in antiferroelectric and ferroelectric orders. The results establish domain walls in noncollinear antiferroelectrics as multifunctional 2D systems with unusual physical properties. Enabled by the symmetry lowering causing the canting of electric dipoles, these 2D systems unify properties that are usually specific to either ferroelectric or antiferroelectric materials, classifying them as hybrid antiferroelectric–ferroelectric (and ferroelastic) walls. The possibility to combine properties that otherwise arise in different types of material—together with the demonstrated tunability—is of interest for the field of domain wall engineering and the design of advanced multifunctional domain-wall-based devices. Furthermore, more advanced structural measurements at the atomic level, such as electron ptychography, could reveal the 3D nature of the walls, their junctions and dynamics, giving additional insights to their hybrid properties. Importantly, the concept of enhancing the physical responses of antiferroelectrics by introducing noncollinearity is not restricted to the case of K_3_[Nb_3_O_6_∣(BO_3_)_2_], and we expect similar effects to arise in a much larger class of materials. Another candidate is the improper ferroelectric Gd_2_(MoO_4_)_3_, where most of the electric dipole moments in the unit cell cancel out and a spontaneous polarization only appears owing to a small canting^12^. Such noncollinear order is symmetry-allowed in all non-centrosymmetric systems with antipolar order in the direction perpendicular to the polar axis. Notwithstanding material-specific details concerning the coupling between polar and antipolar orders, this relationship provides us with a universal guideline where to search for noncollinear antiferroelectricity and noncollinearity-driven nanoscale phenomena as outlined in Supplementary Fig. 20. Noncollinearity may further be leveraged as specific design parameter to enhance the functionality of antiferroelectric heterostructures and multilayers, which already represent versatile host materials for complex dipolar order, including incommensurate and topological antipolar textures^57^, giving a new dimension to the research on antiferroelectrics and anti-ferroic oxides in general.

## Methods

### Macroscopic electric-field switching

*P*(*E*) measurements were performed with a Keithley 6517B electrometer on a (001)-oriented platelet, coated with contacts of silver paint in a customized low-temperature set-up based on a Displex closed-cycle He refrigerator. Owing to experimental limitations, a maximum electric field of 15.9 kV cm^−1^ could be applied. The polarization was measured with two consecutive passes of the electric field (up–down–up–down), and all data shown are hysteresis loops from the second pass, after subtracting a small time-dependent drift of the measured charge.

### Scanning probe microscopy

For all the scanning probe microscopy (SPM) experiments, the samples were polished on a Logitech PM5 polishing machine with a PP5 jig, using Logitech 9 μm calcined aluminium oxide powder as lapping fluid, followed by SF1 polishing suspension, giving a flat surface with a root-mean-square roughness of 696 ± 0.9 pm. For the imaging of a face perpendicular to one of the *a*-axes (inset in Fig. 2a), the sample was cut using a Well 3500 diamond wire saw before polishing.

Lateral PFM was performed with NT-MDT NTEGRA Prima SPM (SFV102NTF head) and an external Stanford SR830 lock-in amplifier, using a diamond-coated TipsNano DCP10 tip with a stiffness of about 15 nN nm^−1^. A 5–10 V a.c. bias at 40.13 kHz was applied to the tip, well below the contact resonance, and the phase offset of the lock-in amplifier was calibrated relative to a poled LiNbO_3_ sample^58^. The contact force during the scans was about 700 nN. The vector PFM procedure is described in Supplementary Note 2.

Vertical PFM, EFM (including temperature-dependent) and localized poling were performed with an Oxford Instruments Cypher ES Environmental SPM, using a Ti/Ir-coated Oxford Instruments ASYELEC.01-R2 tip with a stiffness of about 2.4 nN nm^−1^. For the VPFM, a 5–10 V a.c. bias at contact resonance (about 360 kHz) was applied to the tip. The images were compared with LiNbO_3_-calibrated off-resonance VPFM images, and the phase offset was adjusted accordingly. For the poling, different types of bias were applied to the tip while being in contact with the sample (more details in Supplementary Fig. 15). The contact force during scans and switching was about 100 nN. EFM was performed in dual-pass non-contact mode, with a lift height of 50 nm and a tip d.c. bias of 10 V (no a.c. bias) applied during the second pass.

Tomographic AFM (Fig. 2f) was conducted on the (001) sample face using an Asylum Research MFP-3D Infinity AFM system. The experiment was conducted in two sequential steps. In the first step, a B-doped diamond tip (Adama Technologies) with a stiffness of about 350 nN nm^−1^ was used in the high deflection setpoint regime to remove the material layer by layer. The second step involved use of a Pt/Ir-coated silicon tip (stiffness of about 2.8 nN nm^−1^) to obtain good quality PFM images on the area of interest. These steps were repeated to obtain PFM images at different depths until a final depth of 4 μm was reached. During the tip milling step, the scan size was set to 40 × 40 μm^2^, and PFM at an a.c. frequency of 4 MHz (near contact resonance of the Diamond tip) was performed simultaneously (albeit noisy) to have an overall idea of the domain wall propagation into the depth of the material. After every milling step, clear PFM images were obtained (Supplementary Note 2) through the Pt/Ir-coated tips by applying an a.c. bias of 5–10 V at frequencies near the tip-surface contact resonance, which was around 690 kHz for lateral and 320 kHz for vertical PFM.

### Transmission electron microscopy, specimen preparation and data collection

Tripod wedge TEM specimens were made on an Allied Multiprep system as described in ref. ^59^. Diamond lapping films (DLFs) from 15 down to 0.1 μm grain size were used with a wedge angle of 2°. Final polishing was done with 20 nm silica slurry. No further ion milling was applied. For correlated PLM–PFM–TEM (Supplementary Fig. 11), the specimen was regularly checked with optical microscopy in the final polishing steps until the region of interest was reached. DLFs were used for tripod polishing of TEM specimens. In addition, some specimens were made by focused ion beam (FIB) milling using a Thermo Fisher Scientific G4 UX DualBeam FIB, as outlined in ref. ^60^. In situ lift-out was done with backside milling and a final polishing voltage of 2 kV. (S)TEM was performed with a double spherical-aberration-corrected cold FEG JEOL ARM 200FC, operated at 200 kV. High-resolution HAADF-STEM images were taken with a spatial resolution of 78 pm. HAADF-STEM images included in this work were acquired with a beam semiconvergence angle of 27 mrad, inner and outer semicollection angles of 43 mrad and 170 mrad, and a beam current of 22 pA.

## Online content

Any methods, additional references, Nature Portfolio reporting summaries, source data, extended data, supplementary information, acknowledgements, peer review information; details of author contributions and competing interests; and statements of data and code availability are available at 10.1038/s41565-026-02139-8.

## Supplementary information

Supplementary Information Supplementary Notes 1 and 2, Tables 1 and 2, and Figs. 1–20.

## Source data

Source Data Fig. 2 Source data.

Source Data Fig. 3 Source data.

Source Data Fig. 4 Source data.

Source Data Fig. 5 Source data.

## Data Availability

The source data supporting the findings of this study are available via Zenodo at 10.5281/zenodo.18326068 (ref. ^61^). Source data are provided with this paper.

## Code availability

The code supporting the findings of this study is available via Zenodo at 10.5281/zenodo.18326068 (ref. ^61^).
