# Supplementary material for: Hybrid antiferroelectric–ferroelectric domain walls in noncollinear antipolar oxides
Source: Nat Nanotechnol. 2026 Apr 15;21(5):648–54. doi: 10.1038/s41565-026-02139-8 (PMC13186693; doi:10.1038/s41565-026-02139-8)
Supplement: Supplementary file 1 — Supplementary Notes 1 and 2, Tables 1 and 2, and Figs. 1–20. [file 41565_2026_2139_MOESM1_ESM.pdf]

nature nanotechnology

Supplementary information https://doi.org/10.1038/s41565-026-02139-8

Hybrid antiferroelectric–ferroelectric domain walls in noncollinear antipolar oxides

In the format provided by the authors and unedited

Contents

Supplementary Note 1 **2**

Supplementary Note 2 **4**

Supplementary Tables **6**

Supplementary Figures **8**

Supplementary References **21**

Supplementary Note 1

Density functional theory calculations

For the calculations, we used density functional theory (DFT) within the PBEsol approximation [1] as implemented in VASP [2][3]. The atomic cores were treated using the projector augmented wave approximation [4], considering at least the following electrons explicitly in the calculations: 3p and 4s for K; 4p, 4d and 5s for Nb; 2s and 2p for B; and 2s and 2p for O. The electronic wave functions were described with a plane-wave basis cut off at 500 eV or higher. Reciprocal space integrals – for the high-temperature phase with a 20-atom unit cell – were computed using a grid of at least 3x3x6 k-points in the Brillouin zone (or similarly dense grids when other supercells were considered). Structural relaxations were stopped for residual forces and stresses smaller than 10^{-2} eV/Å and 1 kB, respectively. We note that two different groups (in Norway and Luxembourg) ran DFT simulations in this project, always using calculation conditions that are sufficiently converged, obtaining consistent results.

The piezoelectric and Born tensors were calculated within the framework of density functional perturbation theory (DFPT). $K_3[Nb_3O_6|(BO_3)_2]$ belongs to the 2mm point group and has three independent dielectric tensor components, five independent piezoelectric tensor components, and nine independent elastic stiffness components. The calculated tensor components used for FEM simulations are summarized in Tab. S2.

Finite element modeling

The numerical modeling in Fig. 3(b) in the main text was conducted using a finite element solver integrated into COMSOL Multiphysics (Ver. 6.1). The piezoelectric sample was assumed to be a block with dimensions 500x250x200 nm with the same domain structure as in Fig. 3(a) and planar domain walls. The sample was characterized by assigning $P_a = 0.03$ μC/cm^2, and a complete set of piezoelectric, dielectric, and elastic tensors from Tab. S2. The tip was modeled as a cylinder with a contact radius of 5 nm (value based on the Hertz contact model with experimental parameters) and a height of 1 nm. Mesh refinement in proximity of the tip was conducted sequentially until mesh independence was achieved at approximately 11000 elements. For the elastic equation, the bottom face of the sample was assumed to be fixed. For the electrostatic equation, the lower face of the tip was assumed to have a biased potential of -5 V, whilst the bottom face of the sample was grounded. To simulate the PFM scan operation, a series of simulations were conducted along the path illustrated in Fig. 3(a) in the main text. With this quasi-static approach, the areal average of vertical displacement over the area of the tip's bottom face was calculated in different points of the path.

Supplementary Note 2

Tomographic AFM

We observed no substantial changes (like domain curving, merging, or separating) through the depth of 4 µm. Therefore, three out of the seven images (at 250 nm, 1 µm and 3 µm, Supplementary Fig. S3(a-c)) were chosen to construct Fig. 2(f) in the main text. Firstly, domain walls were isolated from each of the PFM images, then alpha shape triangulation was performed in MATLAB to form a 3D structure.

Additional information: Occasionally, AFM polishing succeeded the milling step to minimize the topographical crosstalk in the imaging stage. In this step, sequential reduction of deflection setpoint was done over a few scans to slowly lower the tip force on the surface and thus smoothening the milled area.

Vector PFM

Vector PFM was performed by doing LPFM on the (001) sample face of $K_3[Nb_3O_6|(BO_3)_2]$ at different sample-cantilever angles (β in Supplementary Fig. S4(a)). The signal strength within a domain is proportional to $\mathbf{P} \cdot \mathbf{v} = P\cos(\beta - \alpha)$, meaning that two domains, e.g., T$'$ and T$''$, would have the same contrast only if the condition $\cos(\beta - \alpha') = \cos(\beta - \alpha'')$ is met. We found three angles β_1, β_2, β_3 (Supplementary Fig. S4(b-d)), at which different domain pairs have the same contrast. The polarization angles α', α'', α''' for the three domains then satisfy:

$$\begin{bmatrix} 1 & 1 & 0 \\ 0 & 1 & 1 \\ 1 & 0 & 1 \end{bmatrix} \begin{bmatrix} \alpha' \\ \alpha'' \\ \alpha''' \end{bmatrix} = 2 \begin{bmatrix} \beta_1 + 180^\circ n_1 \\ \beta_2 + 180^\circ n_2 \\ \beta_3 + 180^\circ n_3 \end{bmatrix} ; \quad (n_1, n_2, n_3) \in \mathbb{Z}^3 \tag{1}$$

We find this equation to have two non-equivalent solutions, consistent with the two possible polarities of the PFM calibration (polarity of vector $\mathbf{v}$). We then find that the inequalities in Supplementary Fig. S4(b-d) satisfy the solution $\alpha' = -60^\circ$, $\alpha'' = 180^\circ$, $\alpha''' = 60^\circ$, which gives us the in-plane polarization directions.

Vertical piezoresponse in bended domain walls

Vertical PFM was performed with the DART (Dual Amplitude Resonance Tracking) mode over a spot with two bended domain walls, shown in Supplementary Fig. S5(a). To enhance image quality, 28 images (7 scans) were averaged, as shown in Supplementary Fig. S5(b). At each scanning line, a cross-section was extracted and its baseline was subtracted with

a node spacing of 10 pixels, and $\pm$ 30 pixels around the peak, as shown in Supplementary Fig. S5(c). The local domain wall intensity was then defined at the absolute maxima of the substracted peak, also defining the position of the domain walls. An example of a baseline substraction from a cross-section in Supplementary Fig. S5(b) is shown in Supplementary Fig. S5(c-d), showing lower absolute intensity at a bended head-to-head domain wall than on a straight tail-to-tail domain wall.

The domain wall angles at each scanning line were then found by smoothing domain wall profiles with a Savitzky–Golay filter and performing numerical differentiation. The intensity was finally plotted against the domain wall angle, as shown in Fig. 5(e-f) in the main text.

Supplementary Tables

Table S1: **Imaginary-frequency phonon modes.** Unstable phonon modes computed at the Γ-point of room temperature supercell. A visualization of these phonon modes for Nb can be found in Supplementary Fig S2.

**Instability #**	**Frequency (i cm^2)**	**Space group**	**Active irrep(s) (primary)**	**Secondary irreps**	P_{tot} **direction**
1	71.260	C1m1 (8)	$\Gamma_6^{(a,0)}$	Γ_1, Γ_3, Γ_5	[0,0,0]
2	71.298	C121 (5)	$\Gamma_6^{(a,a/\sqrt{3})}$	Γ_1, Γ_2, Γ_5	[0,0,0]
3	72.395	P2_1mn (31)	M_3	Γ_1 Γ_5	[0,0,0]
4	72.824	P2aa (27)	LD_2LE_2	Γ_1, Γ_5, M_1	[0,0,0]
5	72.824	P2aa (27)	LD_2LE_2	Γ_1, Γ_5, M_1	[0,0,0]
6[a]	73.429	P2_1ma (26)	LD_3LE_3	Γ_1, Γ_5, M_1	[0,0,0]
7[a]	73.429	P2_1ma (26)	LD_3LE_3	Γ_1, Γ_5, M_1	[0,0,0]
8	73.868	P2na (30)	M_2	Γ_1 Γ_5	[0,0,0]
9	93.963	P2_1mn (31)	M_3	Γ_1 Γ_5	[0,0,0]
10[a]	94.037	P2_1ma (26)	LD_3LE_3	Γ_1, Γ_5, M_1	[0,0,0]
11[a]	94.037	P2_1ma (26)	LD_3LE_3	Γ_1, Γ_5, M_1	[0,0,0]
12[b]	98.087	P31m (157)	Γ_3	Γ_1	[0,0,1]

[a]Instability consistent with observed space group at room temperature.
[b]Instability consistent with double hysteresis loop formation.

Table S2: Numerical values of the non-zero dielectric, piezoelectric, and elastic tensor components in the orthorombic phase of $K_3[Nb_3O_6|(BO_3)_2]$, calculated using DFT. The Voigt convention 4 = 23 = 32, 5 = 13 = 31, 6 = 12 21 is used.

Dielectric tensor		Piezoelectric tensor (pm/V)		Elastic tensor (GPa)	
ϵ_{11}	28.025	d_{11}	1.52	C_{11}	275.3
ϵ_{22}	27.685	d_{12}	-1.48	$C_{12} = C_{21}$	107.1
ϵ_{33}	32.972	d_{13}	-0.011	$C_{13} = C_{31}$	75.3
		d_{26}	-3.195	C_{22}	281.6
		d_{35}	0.84	$C_{23} = C_{32}$	72.5
				C_{33}	157.7
				C_{44}	17.55
				C_{55}	17.65
				C_{66}	86.28

Supplementary Figures

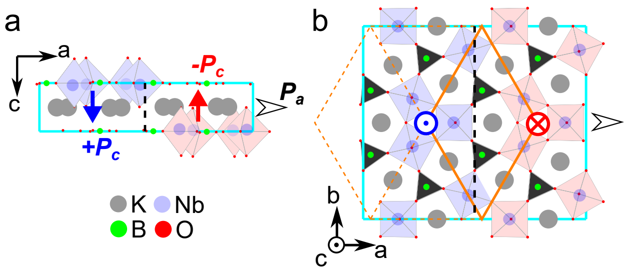

Figure S1: **Room-temperature unit cell of $K_3[Nb_3O_6|(BO_3)_2]$.** a) The canted antipolar arrangement in the *ac*-plane is presented. b) A perfect nearest-neighbor antipolar arrangement of the Nb-trimers in the ab-plane is forbidden by geometry. Antipolar order breaks the threefold symmetry of the high-temperture phase (orange cell), turning the material polar.

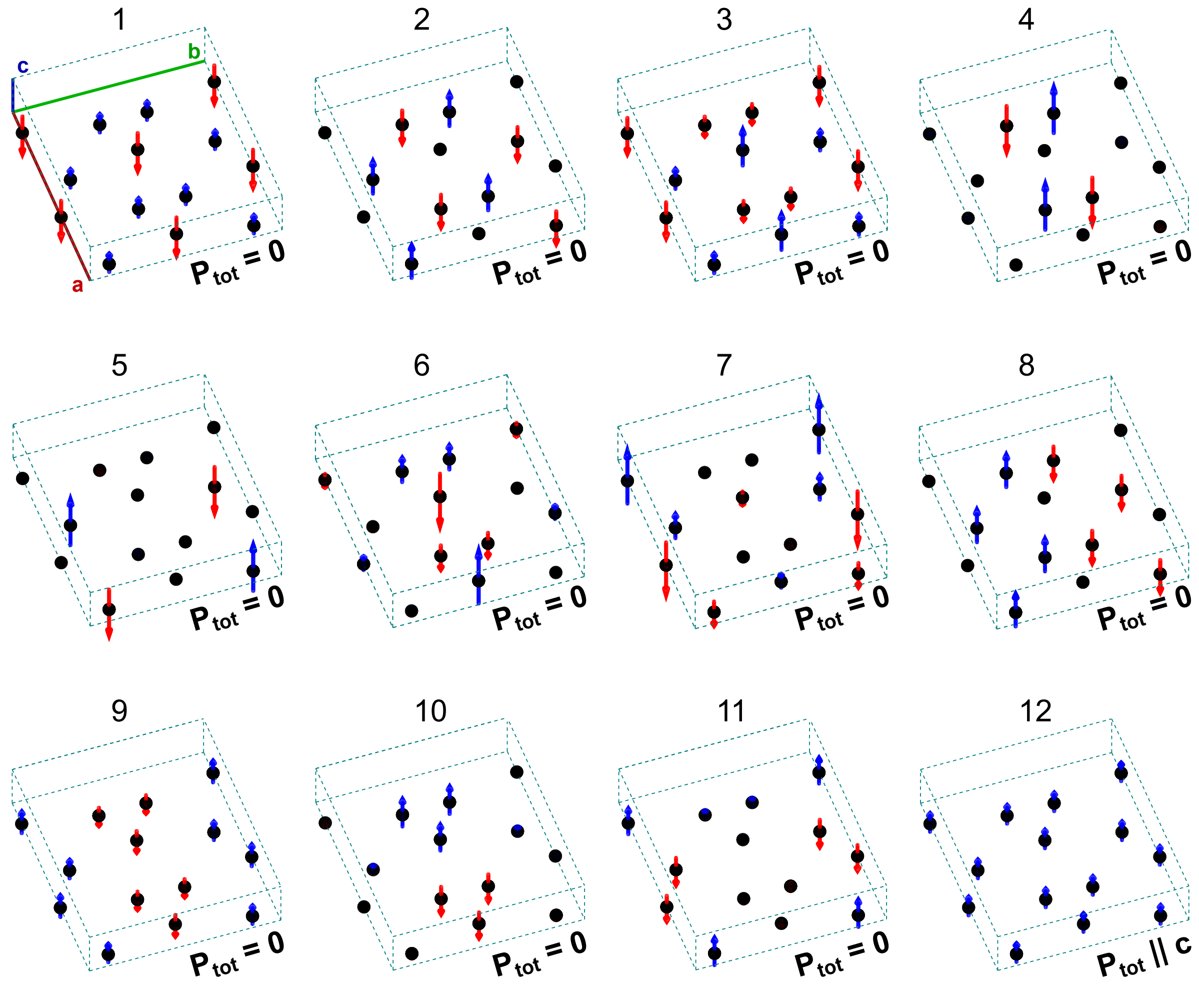

Figure S2: **Nb displacement of phonon modes in Tab. S1.** Nb displacements within the cell of the room-temperature low-symmetry phase. Displacements only happen along the c-axis and, except for case 12, the displacements cancel out so that $P_{tot} = 0$.

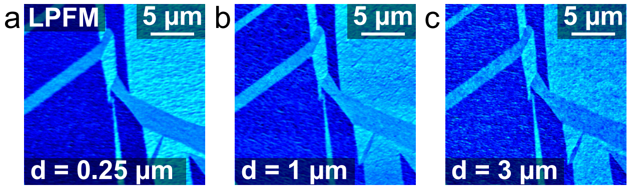

Figure S3: **Cross-sections in tomographic AFM.** a-c) Lateral PFM plotted as $A \cos \phi$ at the three milling depths used for 3D reconstruction in Fig. 2(f) in the main text.

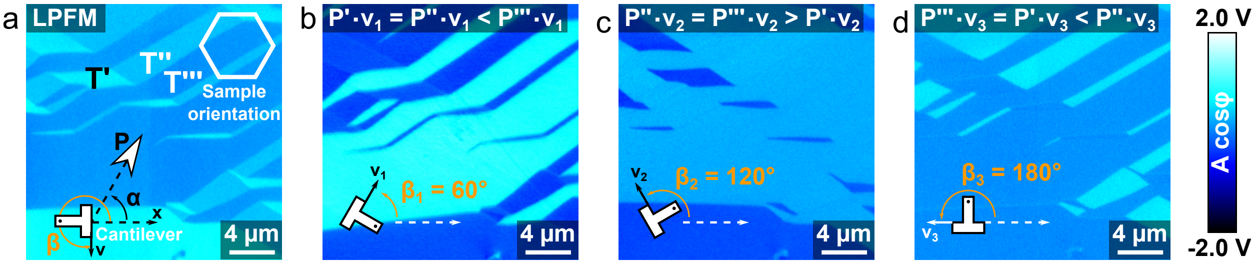

Figure S4: **Vector PFM procedure.** The opposite calibration convention from ref. [5] was used, making the domain signal positively proportional to its in-plane polarization component **P** projected on the vector **v** in a). With three domains in total, we found three sample-cantilever orientations in b-d), where different domain pairs give the same contrast, giving the denoted signal strength equations and inequalities.

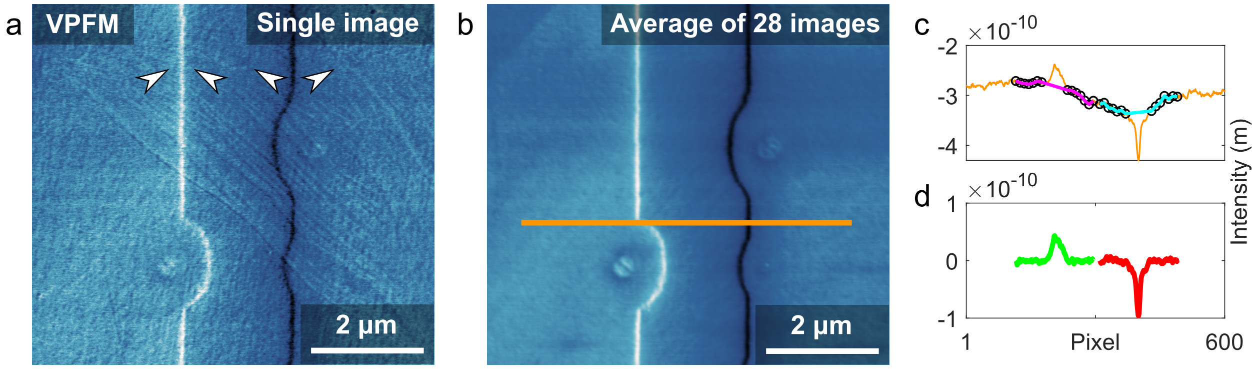

Figure S5: **Piezoresponse of the bended domain walls.** a) Single VPFM image. b) An average of 28 VPFM images. c) Cross-section from b) with baselines for the two peaks. d) Cross-section from c) with baselines subtracted.

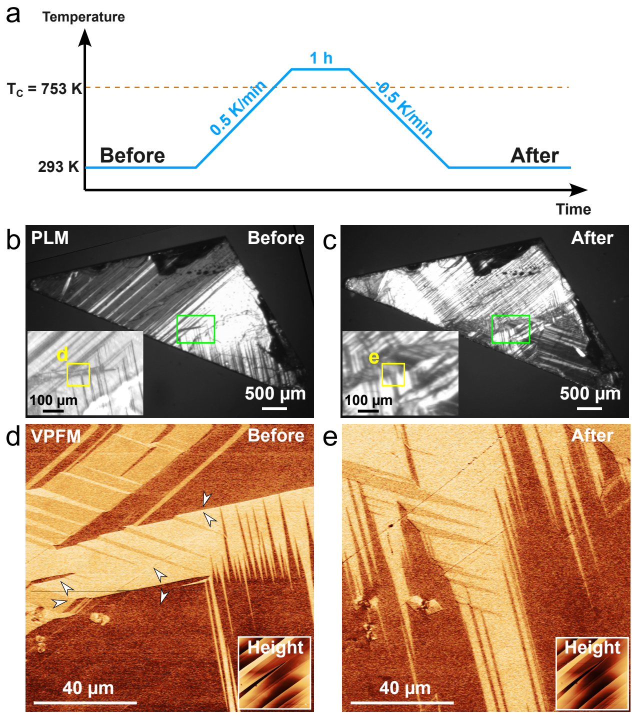

Figure S6: **Domain pattern before and after heating above T_C.** a), Temperature profile showing the concept of the annealing experiment (Synthetic air, Entech Tube furnace). b,c), Polarized light microscopy (PLM) images recorded before (b) and after (c) heating to above T_C. d,e), Piezoresponse force microscopy images (vertical signal, VPFM) taken before (d) and after (e) heating at the positions marked in (b) and (c), respectively. The insets in (d) and (e) show the topography, which was used to identify the same spots for imaging.

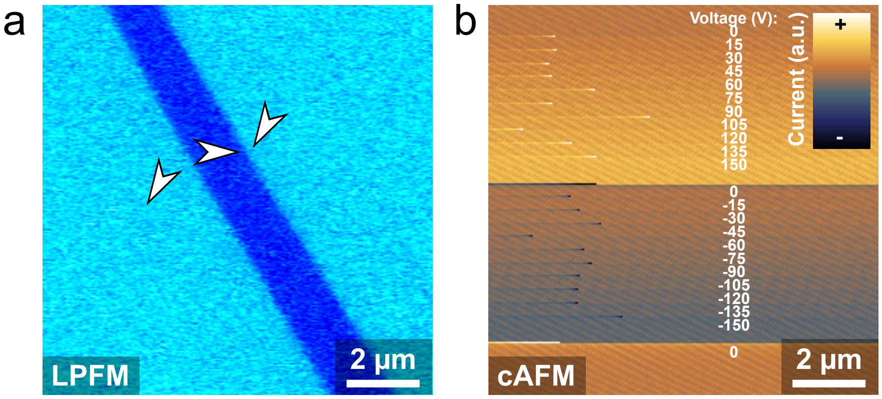

Figure S7: **Conductive atomic force microscopy (cAFM) of hybrid domain walls.** a) Lateral PFM image showing a tail-to-tail and a head-to-head domain wall. b) cAFM of the same spot as in (a) with different bias voltages applied while scanning. The bright and dark spots show current spikes where the voltage was changed, indicating the setup was working properly. No contrast could be observed at the domains and domain walls with voltages up to ±150 V.

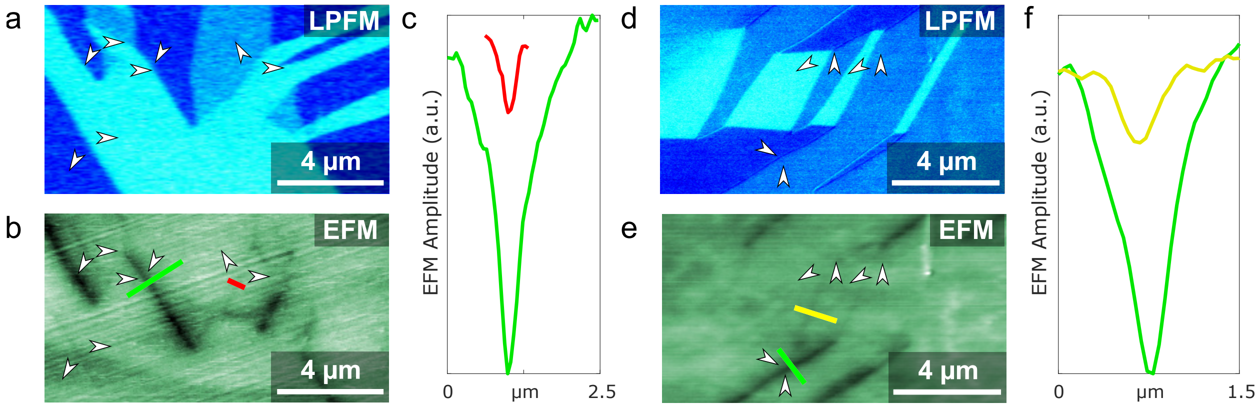

Figure S8: **Electrostatic response at tail-to-tail and head-to-tail domain walls.** a-b) LPFM and EFM of a (001) face region with both head-to-head and tail-to-tail domain walls. For visibility, the rows in b) have been aligned using a one-dimensional polynomial. c) EFM signal of the cross-sections in b), showing that the electrostatic signal measured at head-to-head walls is about 5 times stronger than at tail-to-tail walls. d-e) LPFM and EFM of a (001) face region where some head-to-tail domain walls are visible. f) Cross-sections from e).

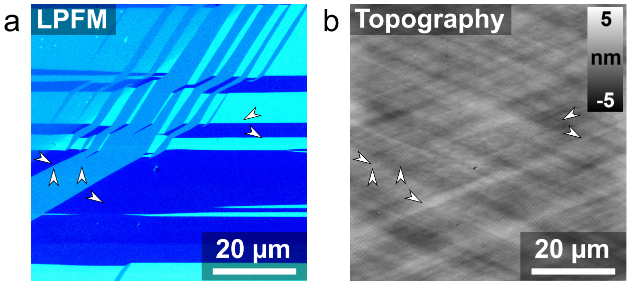

Figure S9: **Topography of polished samples.** a-b) LPFM and AFM, respectively, of the (001) sample face, showing that there are no signatures of domains and domain walls in the topography.

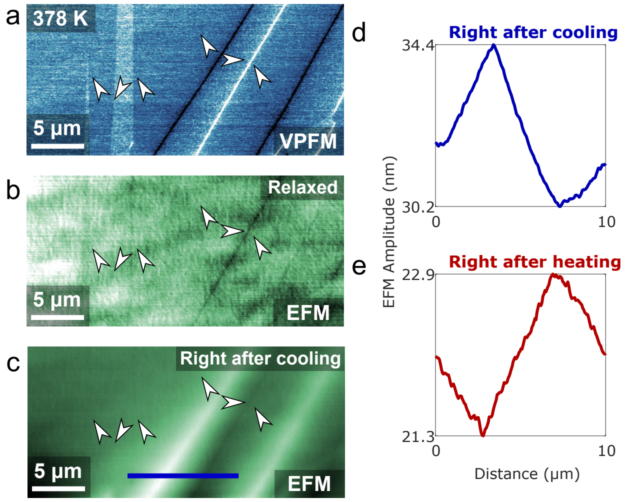

Figure S10: **Electrostatic potential at charged domain walls.** a) Vertical PFM image recorded at 378 K, which is below the reported dielectric anomaly that occurs at about 383-393 K [6]. b) EFM image taken at 378 K at the same spot as in (a) after letting the sample rest for about 30 min (relaxed). The data shows qualitatively the same behavior as observed at room-temperature with the most pronounced EFM response at head-to-head domain walls. c) EFM image of the same spot as in a and b right after cooling from 398 K to 378 K. Here, pronounced contrast is observed at both head-to-head (dark) and tail-to-tail (bright) domain walls. d) Profile from (c). e) EFM scan profile from the same spot as in (d) right after heating from 378 K to 398 K. The results clarify that head-to-head and tail-to-tail walls carry the same amount of charge, consistent with their polar discontinuities ($\pm 2P_a \cos 30^\circ = \pm 0.05\ \mu\text{C/cm}^2$). Differences in surface potential arise only over time, showing that they are extrinsic in nature. Because of the timescale and the reversibility of the process, we attribute the effect predominantly to extrinsic adsorbates which screen the positive and negative domain wall charges with different efficiency [7][8].

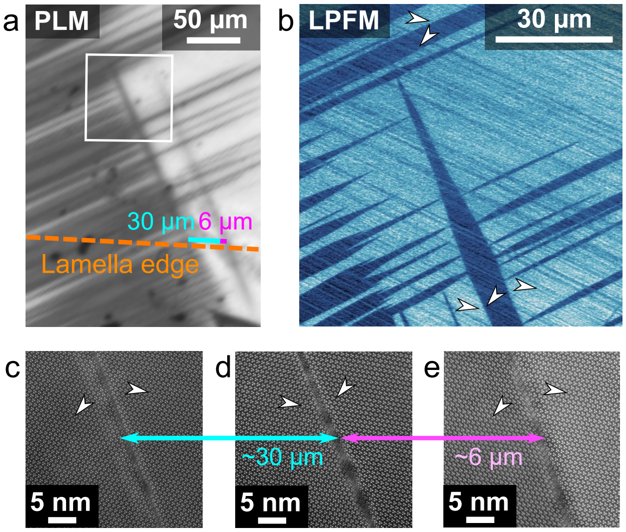

Figure S11: **Correlated PLM-PFM-STEM studies and determination of domain wall types in TEM.** a) PLM image of a (001) sample face before a wedge lamella was prepared. The distance to one needle-like domain and its thickness is shown. b) LPFM of the white square in a), used to determine the polarization directions and the three domain wall types. c-e) Domain walls were observed with the same spacing as shown in a), allowing to determine the type of these domain walls. Note that no head-to-tail walls were observed in TEM, although expected on the lamella edge. Furthermore, the TEM data confirms that the effective shear piezocoefficient is positive with respect to the antipolar canting P_a.

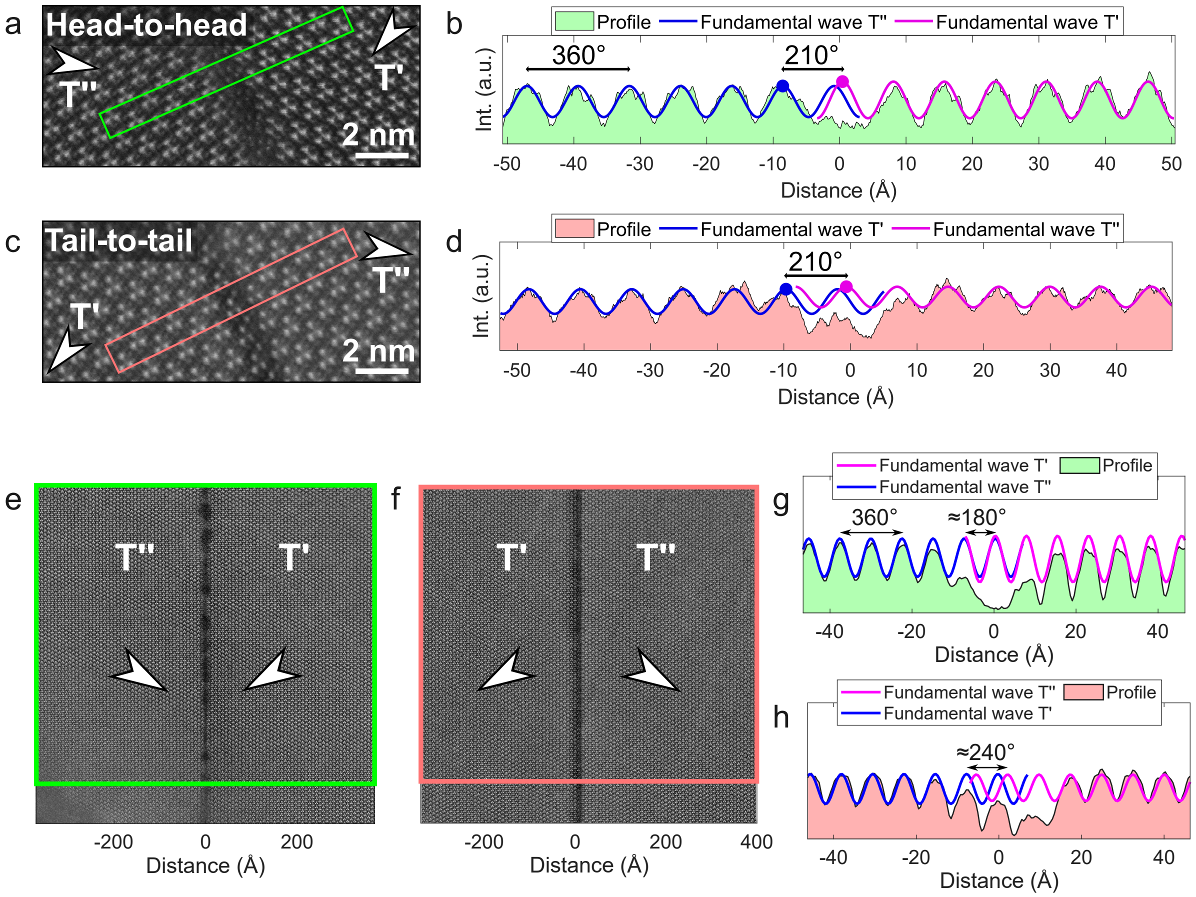

Figure S12: **Structural phase differences at head-to-head and tail-to-tail walls.** a) HAADF-STEM image showing the local structure at representative head-to-head domain wall. b) Profile from (a), with Fourier fit of the fundamental periodicity wave for T" and T' domains, showing a local phase shift of 210°. c,d) same as (a,b) for a tail-to-tail domain wall. e) Large HAADF-STEM scan of a head-to-head domain wall, with green rectangle showing profile averaged along the vertical direction. f) same as (e) for a tail-to-tail domain wall. g,h) same as (b,d) but for profiles in (e-f). The phase shifts approach to different values when the averaging area is increased, consistent with the fact that they need to compensate on larger scales.

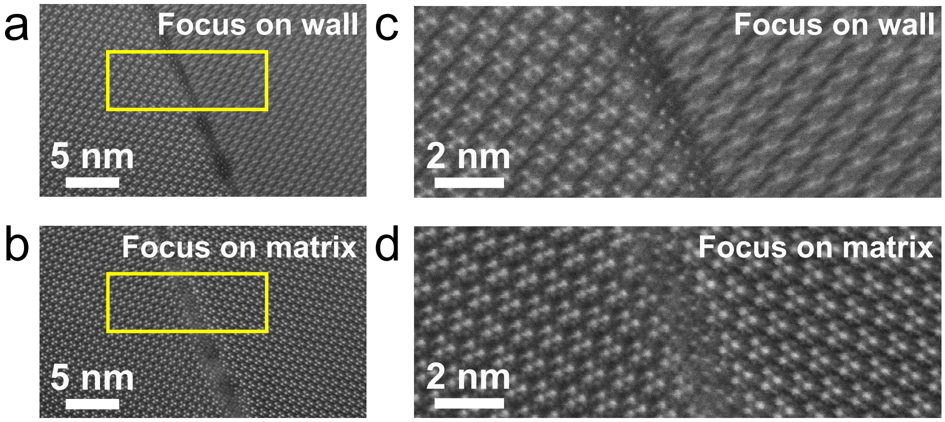

Figure S13: **HAADF-STEM images of a head-to-head domain wall taken with varying focus.** a,c), focus on the wall. b,d), focus on the matrix.

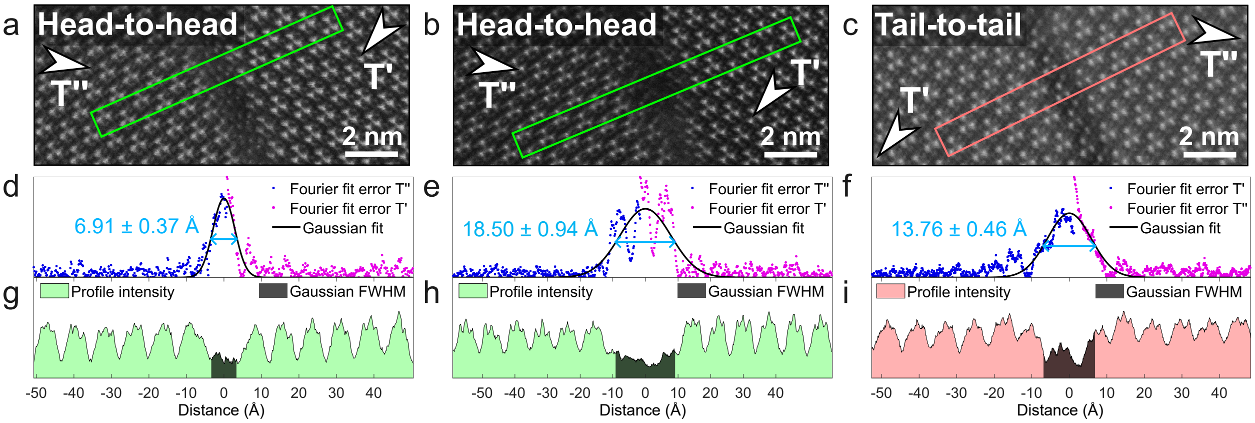

Figure S14: **Domain wall width.** a-c) HAADF-STEM images showing the local structure at representative head-to-head and tail-to-tail domain walls. d-i) To evaluate the domain wall width, we use Fourier analysis, separately fitting the profiles (a-c) on the left and on the right side of the domain walls and then compute the variation across the profile (d-f). The area where the intensity deviates from the bulk values is determined by a Gaussian fit, and the full width at half maximum is then used as a measure/estimate for local domain wall width d_w. For head-to-head walls, we observe local variations of d_w between 6.91 ± 0.37 Å and 18.50 ± 0.94 Å, whereas the investigated tail-to-tail wall has less thickness variations with $d_w = 13.76 \pm 0.46$ Å.

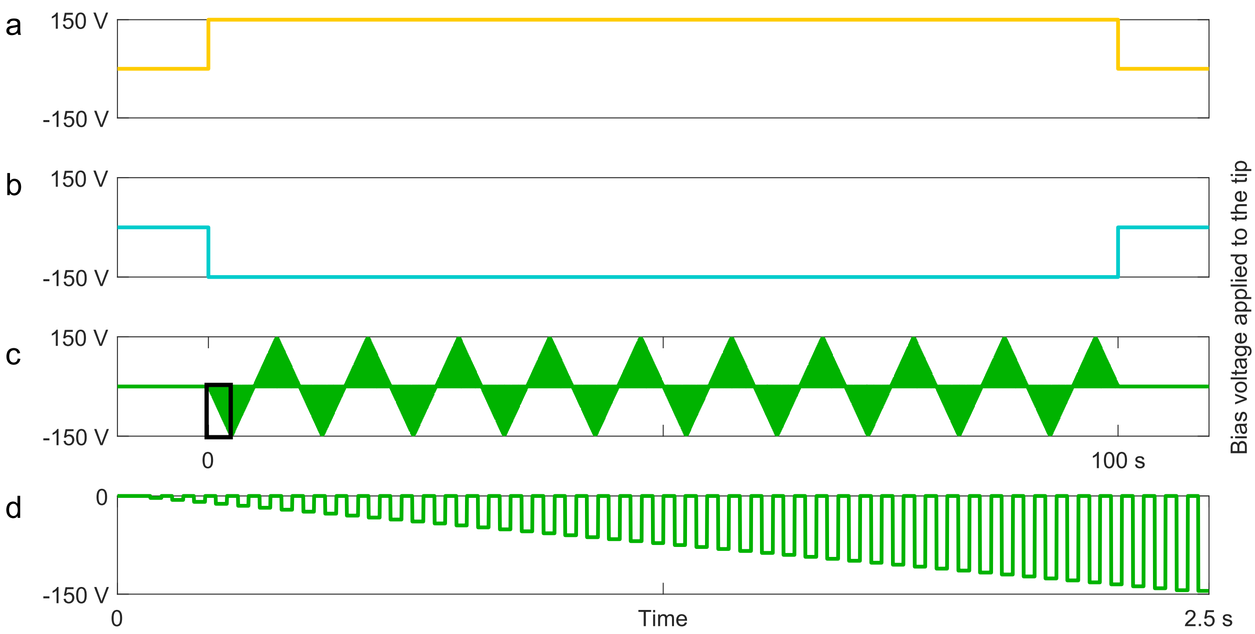

Figure S15: **Bias profiles used for domain wall bending.** a) Positive d.c. bias. b) negative d.c. bias. c) a.c. bias. d) Zoom-in to the black square in c), showing that the a.c. bias is made up of linearly decreasing/increasing step pulses, having 200 steps per period (=10 s) and 10 periods in total.

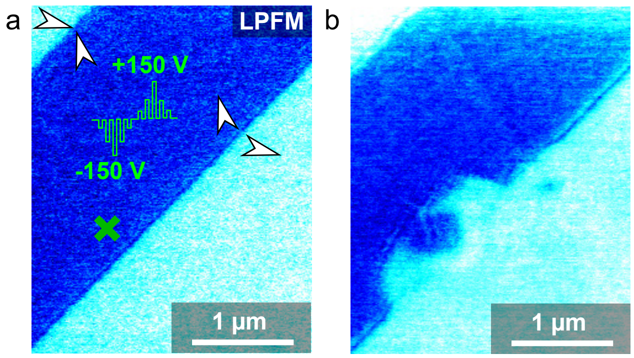

Figure S16: **Domain wall bending.** a-b) Tail-to-tail and head-to-head domain walls move towards their nearest neighbor, as shown here for the case of tail-to-tail wall, even when bias is applied between the walls.

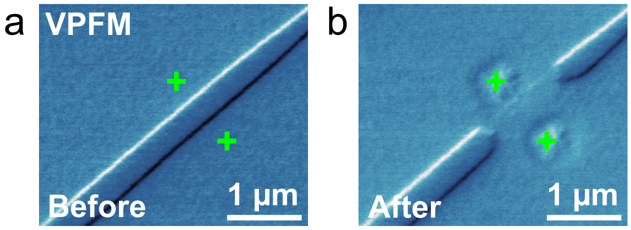

Figure S17: **Annihilation of head-to-head and tail-to-tail domain walls.** The experiment demonstrates that head-to-head and tail-to-tail walls can annihilate during poling, implying that associated phase shifts compensate each other, consistent with previous optical experiments at larger length scales [9].

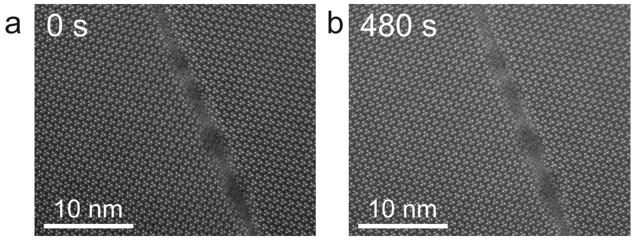

Figure S18: **Time stability of a head-to-head domain wall.** a,b) HAADF-STEM images showing the same segment of a head-to-head wall, taken 480 seconds apart. In between, several exposures were taken under different imaging conditions; no apparent changes are visible due to exposures to the electron beam.

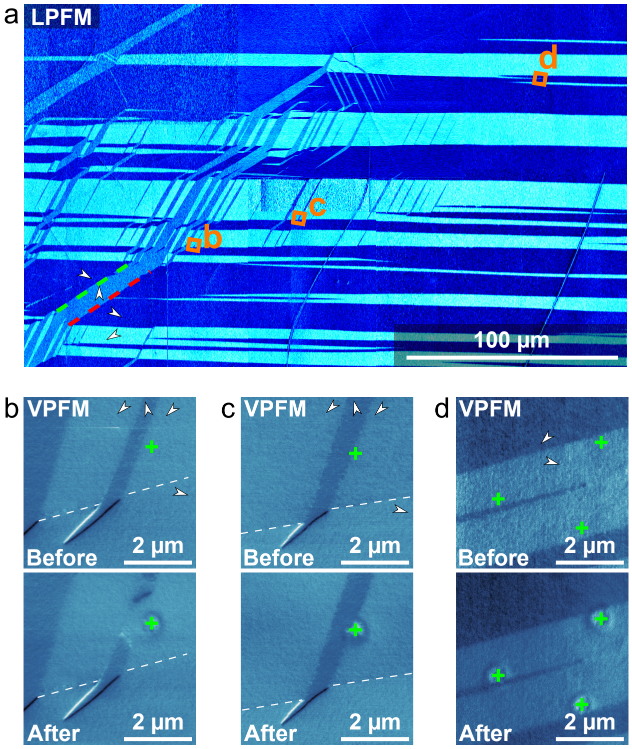

Figure S19: **Switching behavior of head-to-tail domain walls.** a) Stitched LPFM image showing a characteristic domain pattern, including head-to-head and tail-to-tail domain walls marked by green and red dashed lines. Orange squares indicate positions where the poling experiments in (b-d) are performed. b) VPFM images of head-to-tail walls connected to head-to-head (white) and tail-to-tail (black) walls taken before and after applying an a.c. voltage (±150 V) at the marked position. The head-to-tail walls readily move and merge, leading to new charged sections with increased piezoresponse. Repeating the experiment on 16 comparable head-to-tail walls in similar positions shows that 14 walls (87.5 %) move by $\gtrsim$ 200 nm, whereas no substantial displacement ($\lesssim$ 50 nm) is resolved for the rest. c) Same as in (b) recorded further away (≈ 70 µm) from the marked head-to-head and tail-to-tail walls in (a), displaying the case of a head-to-tail wall that remains stationary. Measuring different comparable head-to-tail walls in this regime, we observe that three out of ten (30 %) move under the applied a.c. field. d) Extended experiments on more isolated head-to-tail walls (i.e., away from meeting points and with no head-to-head or tail-to-tail wall nearby) corroborate that they are pinned to their position with only three out of 19 (< 16 %) moving under the applied a.c. field. The experiments (b-d) show a clear trend, indicating that head-to-tail walls connected to head-to-head or tail-to-tail walls switch easier than the more isolated head-to-tail walls, which we attribute to destabilizing structural defects that form at the connection points between head-to-tail and head-to-head or tail-to-tail walls, consistent with the observed phase mismatch between these walls. However, we also find an additional dependence on the distance to coexisting (not connected) head-to-head and tail-to-tail walls, indicating intriguing switching dynamics of the non-conservative domain walls.

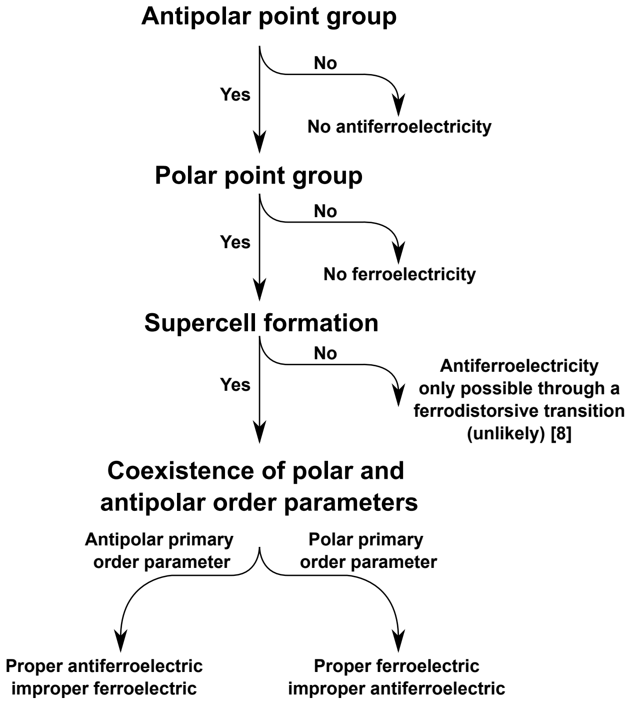

Figure S20: **Identification guideline for hybrid antiferroelectric-ferroelectric materials.** The tree suggests how to screen for related systems, not necessarily covering all possible situations. Note that antiferroelectricity is not defined by symmetry alone, requiring a ferroelectric instability to generate dielectric anomalies and the characteristic double-hysteresis loop [10]. Antipolar and polar point groups can be found, e.g., in refs. [11][12][13], whereas the order parameter analysis can be performed, e.g., using software packages such as ISODISTORT [14] or AMPLIMODES [15].

Supplementary References

[1] Perdew, J.P., Ruzsinszky, A., Csonka, G.I., Vydrov, O.A., Scuseria, G.E., Constantin, L.A., Zhou, X., Burke, K.: Restoring the density-gradient expansion for exchange in solids and surfaces. Physical review letters 100(13), 136406 (2008)

[2] Kresse, G., Furthmüller, J.: Efficient iterative schemes for ab initio total-energy calculations using a plane-wave basis set. Physical review B 54(16), 11169 (1996)

[3] Kresse, G., Furthmüller, J.: Efficiency of ab-initio total energy calculations for metals and semiconductors using a plane-wave basis set. Computational Materials Science 6, 15–50 (1996)

[4] Blöchl, P.E.: Projector augmented-wave method. Physical Review B 50, 17953–17979 (1994)

[5] Soergel, E.: Piezoresponse force microscopy (PFM). Journal of Physics D: Applied Physics 44(46), 464003 (2011)

[6] Kharitonova, E.P., et al.: Polymorphism of ferroelectric, ferroelastic, superionic crystals $K_3Nb_3B_2O_{12}$ and $K_{3-x}Na_xNb_3B_2O_{12}$. Inorganic Materials 38(8), 819–824 (2002)

[7] Kalinin, S.V., Bonnell, D.A.: Local potential and polarization screening on ferroelectric surfaces. Physical Review B 63(12), 125411 (2001)

[8] Kalinin, S.V., Bonnell, D.A.: Screening phenomena on oxide surfaces and its implications for local electrostatic and transport measurements. Nano Letters 4(4), 555–560 (2004)

[9] Voronkova, V.I., et al.: Growth, structure, and properties of ferroelectric—ferroelastic—superionic $K_3Nb_3B_2O_{12}$ and $K_{3-x}Na_xNb_3B_2O_{12}$ crystals. Crystallography Reports 45(6), 816–820 (2000)

[10] Tagantsev, A.K., et al.: The origin of antiferroelectricity in $PbZrO_3$. Nature Communications 4(1), 2229 (2013)

[11] Blinc, R., Žekš, B.: Soft Modes in Ferroelectrics and Antiferroelectrics. North-Holland Publishing Company, Amsterdam, Netherlands (1974)

[12] Shan, P., Long, X.: Symmetry of antiferroelectric crystals crystallized in polar point groups. IUCrJ 9(4), 516–522 (2022)

[13] Zheludev, I.S.: Ferroelectricity and symmetry. In: Solid State Physics, vol. 26, pp. 429–464. Elsevier, Amsterdam, Netherlands (1971)

[14] Campbell, B.J., Stokes, H.T., Tanner, D.E., Hatch, D.M.: ISODISPLACE: a web-based tool for exploring structural distortions. Journal of Applied Crystallography 39(4), 607–614 (2006)

[15] Orobengoa, D., Capillas, C., Aroyo, M.I., Perez-Mato, J.M.: AMPLIMODES: symmetry-mode analysis on the Bilbao Crystallographic Server. Journal of Applied Crystallography 42(5), 820–833 (2009)
